# INFORM2 NivEnt: The first trial of the INFORM2 biomarker driven phase I/II trial series: the combination of nivolumab and entinostat in children and adolescents with refractory high-risk malignancies

**DOI:** 10.1186/s12885-020-07008-8

**Published:** 2020-06-05

**Authors:** Cornelis M. van Tilburg, Ruth Witt, Melanie Heiss, Kristian W. Pajtler, Christoph Plass, Isabel Poschke, Michael Platten, Inga Harting, Oliver Sedlaczek, Angelika Freitag, David Meyrath, Lenka Taylor, Gnana Prakash Balasubramanian, Natalie Jäger, Elke Pfaff, Barbara C. Jones, Till Milde, Stefan M. Pfister, David T. W. Jones, Annette Kopp-Schneider, Olaf Witt

**Affiliations:** 1 KiTZ Clinical Trial Unit, Hopp Children’s Cancer Center Heidelberg (KiTZ), German Cancer Research Center (DKFZ) and Heidelberg University Hospital, Im Neuenheimer Feld 430, 69120 Heidelberg, Germany; 2 Clinical Cooperation Unit Pediatric Oncology, German Cancer Research Center (DKFZ) and German Cancer Consortium (DKTK), Heidelberg, Germany; 3 Hopp Children’s Cancer Center Heidelberg (KiTZ), Department of Pediatric Hematology and Oncology, Heidelberg University Hospital, Heidelberg, Germany; 4 Hopp Children’s Cancer Center Heidelberg (KiTZ), Division of Pediatric Neurooncology, German Cancer Research Center (DKFZ) and German Cancer Consortium (DKTK), Heidelberg, Germany; 5 Division of Cancer Epigenomics, German Cancer Research Center (DKFZ), Heidelberg, Germany; 6 DKTK Immune Monitoring Unit, German Cancer Research Center (DKFZ) and National Center for Tumor Diseases (NCT), Heidelberg, Germany; 7 DKTK CCU Neuroimmunology and Brain Tumor Immunology, German Cancer Research Center (DKFZ), Heidelberg, Germany; 8 Department of Neuroradiology, Heidelberg University Hospital, Heidelberg, Germany; 9 Radiology Cooperation Uni/DKFZ, Division of Radiology, NCT, Heidelberg, Germany; 10 NCT Trial Center, National Center for Tumor Diseases, Heidelberg, Germany; 11 German Cancer Research Center (DKFZ), Heidelberg, Germany; 12 Pharmacy Department, Heidelberg University Hospital, Heidelberg, Germany; 13 Pediatric Glioma Research Group, Hopp Children’s Cancer Center Heidelberg (KiTZ), German Cancer Research Center (DKFZ) and German Cancer Consortium (DKTK), Heidelberg, Germany; 14 Division of Biostatistics, German Cancer Research Center (DKFZ), Heidelberg, Germany

**Keywords:** Entinostat, Nivolumab, Child, Bayesian design, Biomarker, Phase I/II, Checkpoint inhibition, HDAC

## Abstract

**Background:**

Pediatric patients with relapsed or refractory disease represent a population with a desperate medical need. The aim of the INFORM (INdividualized Therapy FOr Relapsed Malignancies in Childhood) program is to translate next generation molecular diagnostics into a biomarker driven treatment strategy. The program consists of two major foundations: the INFORM registry providing a molecular screening platform and the INFORM2 series of biomarker driven phase I/II trials. The INFORM2 NivEnt trial aims to determine the recommended phase 2 dose (RP2D) of the combination treatment of nivolumab and entinostat (phase I) and to evaluate activity and safety (phase II).

**Methods:**

This is an exploratory non-randomized, open-label, multinational and multicenter seamless phase I/II trial in children and adolescents with relapsed / refractory or progressive high-risk solid tumors and CNS tumors. The phase I is divided in 2 age cohorts: 12–21 years and 6–11 years and follows a 3 + 3 design with two dose levels for entinostat (2 mg/m^2^ and 4 mg/m^2^ once per week) and fixed dose nivolumab (3 mg/kg every 2 weeks). Patients entering the trial on RP2D can seamlessly enter phase II which consists of a biomarker defined four group basket trial: high mutational load (group A), high PD-L1 mRNA expression (group B), focal MYC(N) amplification (group C), low mutational load and low PD-L1 mRNA expression and no MYC(N) amplification (group D). A Bayesian adaptive design will be used to early stop cohorts that fail to show evidence of activity. The maximum number of patients is 128.

**Discussion:**

This trial intends to exploit the immune enhancing effects of entinostat on nivolumab using an innovative biomarker driven approach in order to maximize the chance of detecting signs of activity. It prevents exposure to unnecessary risks by applying the Bayesian adaptive design for early stopping for futility. The adaptive biomarker driven design provides an innovative approach accelerating drug development and reducing exposure to investigational treatments in these vulnerable children at the same time.

**Trial registration:**

ClinicalTrials.gov, NCT03838042. Registered on 12 February 2019.

## Background

Children and adolescents with relapsed or refractory malignant disease of a high-risk entity have a particularly poor prognosis. Survival rates of less than 20% after recurrence [1–12] imply an urgent need for innovative treatment strategies. The aim of the INFORM (INdividualized Therapy FOr Relapsed Malignancies in Childhood) program is to translate next generation molecular diagnostics into a personalized, biomarker driven treatment strategy. The program consists of two major foundations: the INFORM registry (https://www.dkfz.de/en/inform/index.html) providing a molecular screening platform [13, 14] and the INFORM2 series of biomarker driven phase I/II trials of which the trial protocol described here was the first to receive Voluntary Harmonisation Procedure plus (VHP+) approval by the European competent authorities.

Cancer immunotherapy was designated as breakthrough of the year already in 2013 [15]. Although promising responses to checkpoint inhibition have been reported in pediatrics, e.g. with nivolumab in hypermutated glioblastoma multiforme [16], pediatric oncology patients in general seem less responsive to checkpoint inhibition. The reason is probably that compared with adult cancers most pediatric cancers carry relatively few mutations that can act as neo-antigens. Adult cancers are frequently driven by chronic mutagenic exposure from environmental factors [17, 18]. Thereby, pediatric cancers are probably less immunogenic than most of their adult counterparts. Histone deacetylase (HDAC) inhibition (HDACi) modifies T-cell regulation [19–21] and can augment response to checkpoint inhibition by reducing the number of myeloid-derived suppressor cells [22] and creating an immunogenic tumor microenvironment including induction of major histocompatibility complex and neo-antigens [23]. Moreover, HDACi induce cryptic transcriptional start sites resulting in several hundred potentially immunogenic transcripts in cancer cells [24]. A phase II study of non-small cell lung cancer patients previously progressing or refractory on anti-PD-1/programmed death-ligand 1 (PD-L1) treatment showed responses to combination of entinostat plus pembrolizumab [25]. Similarly, addition of entinostat to pembrolizumab in anti-PD-1/PD-L1 refractory melanoma patients resulted in objective responses [26]. Both studies suggest that HDACi can enhance the activity of immune checkpoint inhibitors.

In INFORM2 NivEnt, the combination treatment of nivolumab and entinostat is applied in children for the first time, and moreover, in biomarker enriched strata. The phase I will determine the recommended phase 2 dose (RP2D) of the combination and will be seamlessly followed by a biomarker defined four group (A - D) phase II basket trial. The groups A – D in phase II are defined by the following rationales:

- Group A: tumors with a high mutational load (> 100 somatic single-nucleotide variants/exome)
  In addition to a small fraction of pediatric tumors with a true hypermutated phenotype in the context of constitutional mismatch repair deficiency, sporadic tumors can also carry a high mutational load. In a meta-analysis of trials across various adult cancer types treated with anti-PD1/PDL1-Ab as single agent, a low tumor mutational burden of ≤5/megabase (MB) (approximately corresponding to < 100 single-nucleotide variant (SNV)/exome) was associated with 12% response rate versus medium/high mutational burden of 6/MB or greater (corresponding to > 100 SNV/exome) associated with 43% response rate [27]. Within the INFORM registry molecular diagnostic pipeline ~ 6% of patients across a variety of entities show a mutational burden of > 100 somatic missense SNVs/exome. Thus, group A includes sporadic patients with a high mutational load (> 100 somatic missense SNVs/exome) in addition to patients with true hypermutation.
- Group B: high PD-L1 mRNA expressing tumors
  Increased PD-L1 expression is associated with clinical responses to nivolumab in adult non–small-cell lung cancer [28]. Within the INFORM registry molecular diagnostic pipeline ~ 7% of patients across a variety of entities show an increased PD-L1 mRNA expression (CD274: defined as reads per million total reads per kilobase of exon model (RPKM) by RNA-Seq > 3), independent from the high mutational load phenotype.
- Group C: tumors with high-level MYC or MYCN (MYC(N)) amplification
  Very compelling recent preclinical data strongly suggests that HDACi are active against MYC(N) amplified tumors [29–32]. In addition, MYC is reported to upregulate PD-L1 [33]. Within the INFORM registry molecular diagnostic pipeline ~ 5% of patients across a variety of entities show MYC(N) amplification. The broad immunological effects of entinostat which can synergize with checkpoint inhibition will therefore be exploited in this group of very aggressive tumors in addition to a direct inhibiting effect on MYC(N) function and expression.
- Group D: tumors with a low mutational load, low PD-L1 mRNA expression and no MYC(N) amplification
  This “biomarker low” group allows exploring activity in patients with tumors harboring a low mutational burden/PD-L1 expression/no MYC(N) amplification. Because of the above described immune enhancing activity of entinostat, it is postulated that these patients may also benefit from the combination treatment.

## Methods/design

### Study design

**Fig. 1:**
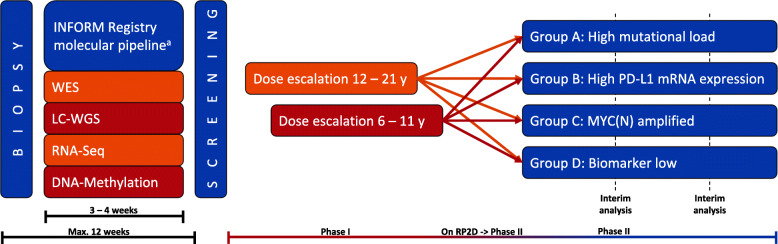
Schematic study overview. Figure shows the workflow of the INFORM Registry molecular pipeline followed by the respective phase I and phase II of the INFORM2 NivEnt trial. WES, whole exome sequencing; LC-WGS, low coverage whole genome sequencing; RNA-Seq, RNA sequencing. ^a^ Or equivalently valid molecular pipelines

INFORM2 NivEnt is an exploratory non-randomized, open-label, multinational and multicenter seamless phase I/II trial of nivolumab and entinostat in children and adolescents with relapsed / refractory or progressive high-risk solid tumors and CNS tumors. A schematic overview of the trial is provided in Fig. 1. Since the age spectrum comprises a broad range of physical development of the study patients, phase I is divided in 2 age cohorts: 12–21 years and 6–11 years (in the absence of a pediatric liquid formulation for entinostat, no patients younger than 6 years can be enrolled). Although one could argue that for adolescents no dose escalation is necessary since there is no biological reason to expect another RP2D [34], it was decided to include a dose escalation for adolescents since the available adult data is still immature. The separate dose escalations for children 6–11-year-old and adolescents 12–21-year-old was included to fulfill regulatory requirements. The two age cohorts will run independently and follow a 3 + 3 design with two dose levels [35]. With only two dose levels, a model-based phase I design such as the Continual Reassessment Method was considered but would not offer any advantage to the traditional 3 + 3 design. RP2D is the dose at which up to 1 of 6 patients experiences dose limiting toxicity (DLT). Assuming more similarity with adult data, the trial will start with the older age cohort. The younger age cohort starts if either of the following events occur: 0 of 3 patients of the older age cohort experience a DLT in the DLT observation period OR a maximum of 1 of 6 patients of the older age cohort experience a DLT in the DLT observation period. Patients entering the trial on RP2D can seamlessly enter phase II. In order to maximize the chance of detecting early signs of activity of the combination treatment, phase II consists of a biomarker defined four group (A - D) phase II basket trial. If patients harbor more than one biomarker fitting group A, B or C, they will be allocated according the following priority: A: highest, B: intermediate and C: lowest priority. The aim is to identify the biomarker cohorts in which the combination treatment shows promising activity, i.e. a response rate exceeding the historical response rate of p_0_ = 12% which was observed in a comparable pediatric patient population treated in phase I/II trials [3]. A response rate of p_1_ = 30% would be considered as the target response rate to be detected with high probability.

**Fig. 2:**
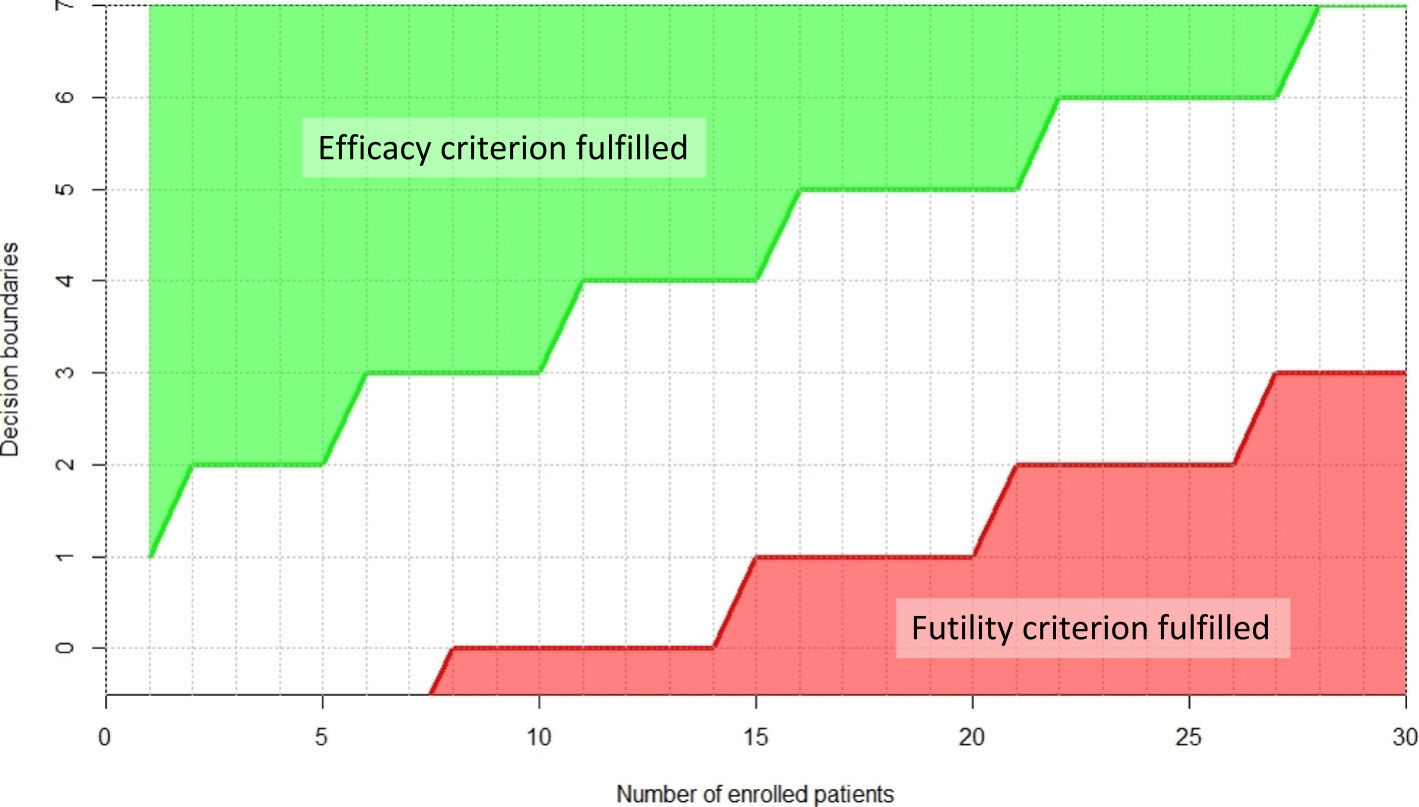
Decision boundaries for futility and efficacy in terms of number of observed responders among enrolled patients. The same criteria apply for interim and final analyses

A Bayesian adaptive design will be used with the objective of stopping cohorts early which are showing no evidence of activity (stopping for futility) [36–38]. Interim analyses will be based on the Bayesian posterior probability distribution of the best response rate, r. Futility will be declared if the posterior probability that r is ≥ p_1_ is smaller than c_Futility_. Activity will be declared if the posterior probability that r is ≥ p_0_ is larger or equal to c_Activity_. The Bayesian model will be based on the Beta-binomial conjugate family. For futility, the prior is chosen as an optimistic Beta(p_1_, 1-p_1_), and for activity the choice is accordingly a sceptical Beta(p_0_, 1-p_0_). Every cohort will be evaluated separately. An interim analysis is planned after 10 patients. If recruitment allows, further interim analyses are planned every 10 patients. Based on the evaluation of the operating characteristics of the trial design, c_Futility_ was selected as 0.01 and c_Activity_. as 0.90 [37]. The resulting decision rules for futility and efficacy in terms of number of observed responders among enrolled patients are shown in Fig. 2. The probability of stopping for futility at first interim when the treatment is not active is at least 27.8% whereas it is only 2.8% for an active treatment with true response rate of 30%.

Three scenarios are possible following interim analysis:

- In case the efficacy criterion is met, no early stopping is planned. However, early evidence of efficacy may set in motion the conception of a subsequent phase II/III trial.
- In case neither the efficacy nor the futility criterion is met, recruitment will continue.
- In case the futility criterion is met, recruitment of patients will be suspended and the DMC will be notified. The DMC will advise the coordinating investigator whether to terminate or to amend the trial.

### Study objectives

#### Primary objective phase I

To determine the RP2D of the combination treatment with nivolumab and entinostat administered to adolescents 12–21 years and children 6–11 years with progressive, relapsed, refractory high-risk solid tumors and CNS tumors.

#### Primary objective phase II

To evaluate activity and safety of the combination treatment with nivolumab and entinostat in children and adolescents with refractory/relapsed/progressive high-risk solid tumors and CNS tumors in the four different biomarker defined groups A – D.

#### Secondary objectives

- Comparison of patient outcomes in group D (biomarker low) with all biomarker positive groups A, B and C (pooled and separately)
- Comparison of patient outcomes in group A-D with matching groups of the INFORM registry (pooled and separately)
- Evaluation of somatic SNV count as a predictive biomarker: relation of patient outcomes to the level of somatic SNVs
- Evaluation of PD-L1 mRNA expression as a predictive biomarker: relation of patient outcomes to the level of PD-L1 mRNA expression
- Evaluation of the level of MYC(N) amplification as a predictive biomarker: relation to patient outcomes
- Evaluate activity using immune related response evaluation methods
- Entinostat plasma PK (cerebrospinal fluid (CSF) if appropriate (e.g. if CSF has to be obtained for clinical reasons))

#### Exploratory objectives

- Exploration of molecular resistance/relapse mechanisms
- Exploration of relevant germline and somatic variants of pharmacogenes (absorption, distribution, metabolism and excretion (ADME)) and their association with treatment response, adverse events and drug metabolism
- Exploration of response prediction of a co-clinical patient derived xenograft (PDX) model and drug testing program
- Explore prediction of neoantigens in relation to SNV counts
- Evaluate the correlation of the level of PD-L1 expression by immunohistochemistry with mRNA expression and patient outcomes
- Evaluate phenotype and function of peripheral immune cells and response to treatment as a predictive biomarker
- Exploration of tumor infiltrating immune cell populations by mRNA expression as a potential predictive biomarker for response
- Exploration of gene signatures as potential predictive biomarkers for response
- Exploration of activation of cryptic transcription start sites as a predictive biomarker
- Exploration of the role of aneuploidy in response to the combination treatment
- Exploration of the role of CD28/B7 costimulatory pathway in response to the combination treatment
- Exploration of the possible role of circulating tumor DNA for monitoring of therapy response and resistance

### Trial end points

#### Primary end points phase I (outcomes)

##### DLT of the combination treatment

Definition of DLT: A DLT is defined as any adverse event (AE) according to the definitions and exceptions listed below that is related (Defined as that there is a reasonable causal relationship between study drug administration and the AE) to the administration of the combination of investigational agents occurring during the priming week and first cycle of combination treatment (first 5 weeks) in phase I of the trial.

A study participant will be considered evaluable for a DLT if at least 2 doses of nivolumab and 4 doses of entinostat were administered during the first 5 weeks (5 weeks normally incorporate the priming week and 1 cycle of planned combination treatment). Participants who discontinue treatment or have treatment delays preventing them from receiving the above defined minimal amount of treatment in the first cycle of combination treatment for reasons unrelated to study drug toxicity, are not evaluable for DLT and will be replaced in enrollment (maximum number of replacement subjects will be 3 per dose level). In case of doubt, the steering committee will determine whether toxicity qualifies as DLT. In addition, the steering committee will consider drug related AEs occurring after the DLT period (first cycle) in assessment of overall tolerability.

The following drug-related AEs will be considered DLT:

- Grade ≥ 3 AE of any duration, please see exceptions below.
- Any immune mediated adverse event, regardless of grade, which requires discontinuation of study treatment (e.g. pneumonitis, hypersensitivity reaction, acute kidney injury) will be a DLT.
- A cumulative delay of ≥14 days of combination treatment for reasons of toxicity during the DLT observation period (first 5 weeks), please see exceptions below.
- Aspartate aminotransferase (AST), alanine aminotransferase (ALT) or total bilirubin Grade ≥ 3 elevation of any duration.
- Grade 4 laboratory values included in the visit schedule of any duration (not listed in the exceptions below).

The following drug-related AEs will NOT be considered DLT:

- Laboratory value AEs:
- Leukopenia, anemia and neutropenia: resolved to Grade 2 within 14 days (unsupported).
- Alkaline phosphatase asymptomatic Grade 3 elevation of any duration.
- Electrolyte abnormalities of any Grade resolving to Grade 1 ≤ 7 days (independent of intervention).
- Lipase or amylase asymptomatic increase of any Grade and duration.
- Lymphocytopenia of any Grade and duration.
- All other Grade ≤ 3 laboratory values resolved to Grade 0–1 within 14 days.
- Other AEs:
- Grade ≤ 3 neurologic AE consistent with immune-treatment effect (i.e., due to peri-tumoral edema/reaction in brain tumors) and resolved to Grade 0–1 within 14 days (with appropriate treatment).
- Fever or vomiting lasting less than 3 days.
- Fatigue: resolved to Grade 2 within 14 days.

Frequency distributions of DLTs will be provided.

#### Primary end point phase II (outcomes)

Best response (CR or PR) will be based on Response assessment in neuro-oncology (RANO) criteria for all primary CNS tumors and Response evaluation criteria in solid tumors (RECIST) for non-CNS tumors, defined for each patient as the best response under study combination therapy during the first 6 cycles (assessment every 2 cycles) by central review. The proportion of responders will be reported as response rate.

Calcified or intra-osseous (osteo) sarcoma target lesions which were progressive before initiation of treatment and show SD on response evaluation (confirmation through a subsequent scan at least 4 weeks later) will be considered as a responder.

#### Secondary end points (outcomes)

- Duration of Response (DOR).
- Disease Control Rate (DCR).
- Stable disease (SD).
- Progression-free survival (PFS).
- Time to Response (TTR).
- Overall Survival (OS).
- Immune related Response Rate (RR) measured by immune-related RECIST (iRECIST) criteria and immunotherapy RANO (iRANO) criteria by central review.
- Entinostat plasma PK.

#### Exploratory end points (outcomes)

- Assessment of circulating tumor DNA in peripheral blood at baseline and at every response evaluation.
- Response prediction in a co-clinical PDX model and drug testing program.
- Immune phenotyping (FACS panel) and Luminex cytokine panel in peripheral blood at baseline, after the priming week and after 5 weeks of initiation of therapy.
- Analyze mRNA expression data for tumor infiltrating immune cell populations.
- Analyze gene signatures in whole exome data.
- Test induction of cryptic transcription start sites at baseline, after priming and after 5 weeks of initiation of therapy.
- Determination of SNV load by different methods (WES, Panel-Seq).

### Trial participants

Recruitment and treatment of patients will be performed in 5 Society of Pediatric Oncology and Hematology (GPOH) academic phase I/II centers in Germany and additional ITCC (http://www.itcc-consortium.org/) academic centers in Stockholm (Sweden), Utrecht (The Netherlands), Paris (France) and Vienna (Austria) together with the academic centers in Sydney, Melbourne and Perth (Australia). An up to date full list of study sites can be obtained on ClinicalTrials.gov, NCT03838042. The protocol (available on request) was or will be approved by all respective competent authorities and ethics committees at all participating institutions, and written informed consent will be requested from all patients and/or legal representatives.

Key inclusion criteria include children and adolescents with refractory/relapsed/progressive high-risk solid and CNS tumors with no standard of care treatment available, age ≥ 6 to ≤21 years. Determination of biomarkers for patient stratification into group A - D is not part of this trial and will be performed in laboratories complying with DIN EN ISO/IEC 17025 or similar. For several countries, biomarkers are routinely determined according the INFORM registry molecular pipeline [13], which has been technically validated. Equivalently valid molecular pipelines are accepted. The following methods are routinely applied as described previously [13]: whole exome sequencing, low coverage whole genome sequencing, RNA sequencing, and DNA methylation array [39]. To try to ensure that molecular profiling matches the current tumor episode as closely as possible (i.e. to minimize the time in which tumor molecular characteristics may have evolved), enrollment within 12 weeks (this also includes the time for the molecular analyses and allows patient transfer to trial sites, wash out etc.) after biopsy/puncture/resection is requested (see Fig. 1). A complete overview of the inclusion- and exclusion criteria is outlined in the Additional file 1.

### Dosing and dose adjustments

In this trial, nivolumab will be administered using the approved dose for adults, which is also the pediatric RP2D (3 mg/kg every 2 weeks) [40]. An ongoing phase I trial with entinostat monotherapy in children identified a RP2D of 4 mg/m2 weekly [41]. The recommended adult dose regimen is 5 mg weekly – 10 mg every 2 weeks (which equals 2.9 mg/m2 weekly – 5.8 mg/m2 every 2 weeks). The combination of entinostat, nivolumab ± ipilimumab was considered safe and tolerable in adults [42]. In the phase I part of the trial, entinostat has 2 dose levels: 2 mg/m2 and 4 mg/m2 (the latter reflecting the single agent pediatric RP2D). The dose escalation will be performed separately in both age groups.

After one so called priming week (to “prime” the immune system with entinostat before applying checkpoint inhibition) with 1 dose of entinostat on day 1, cycles of 4 weeks each will start with entinostat (orally) on day 1, 8, 15 and 22 and intravenous administration of nivolumab on day 1 and 15. Thereafter, every cycle follows the same schedule. There is no pause between cycles. All groups A – D will receive the same treatment.

The toxicity profiles of nivolumab and entinostat show a large overlap. Therefore, AEs will always be managed first by delaying nivolumab and entinostat administration together in combination with symptomatic treatment:

- Phase I: After the occurrence of a DLT in phase I in dose level 2, it is allowed to de-escalate to dose level 1 (see Additional file 2) intra-individually if this is conform the dose-adjustment rules (provided in Additional file 3). If phase I patients do not tolerate dose level 1, treatment should be discontinued.
- Phase II: The dose de-escalation scheme is depicted in Additional file 2. For patients in dose level 1 it can be considered to de-escalate to nivolumab monotherapy (dose level − 1). If this is also not tolerated, treatment should be discontinued. Treatment modifications for toxicity are described in Additional file 3.

### Assessments and collection of outcomes

An overview about diagnostic and therapeutic measures, timing of disease assessment, and study visits is displayed in Additional file 4. In brief, routine visits are planned weekly during the first 2 cycles and every 2 weeks thereafter. Response assessments are scheduled every 8 weeks.

#### Data collection

All findings including clinical and laboratory data will be documented by the investigator or an authorized member of the study team in the patient’s medical record and in the electronic case report form (eCRF). The eCRF will be created by using the electronic data capture System MARVIN by XClinical, which is used and established within the GPOH and available in all participating centers. All data will be reported and collected pseudonymized.

#### Primary outcome phase I

The definition for the phase I primary outcome DLT of the combination treatment is described above. Planned times for all safety assessments are listed in the visit plan in Additional file 4. Safety assessments include AEs (on the basis of the 5-grade scale defined in the Common Terminology Criteria for Adverse Events (CTCAE) v5.0), physical examinations, vital signs, weight, performance status, assessment of signs and symptoms, laboratory tests, ECGs and pregnancy tests.

#### Primary outcome phase II

The phase II primary outcome best response will be assessed at each follow-up scan prospectively according to the visit plan in Additional file 4. Study endpoints of response or progression will be based on RANO criteria for all CNS tumors [43] and RECIST for all solid tumors. Further disease specific response criteria can be applied in individual cases to the investigator’s discretion. MRI is the preferred modality, but alternative/additional modalities can be used if this is required for certain entities according to institutional or standard of care guidelines. All radiologic imaging will be submitted for blinded independent central review in a pseudonymized fashion at the study’s reference radiology within 2 working days. Importantly, progressive disease will only be confirmed after central review. As a secondary endpoint and in accordance with Borcoman et al. [44], for patients who continued treatment beyond progression in case of clinical benefit, response as assessed by iRECIST [45] or iRANO [46] will be performed.

### Sample size

Phase I (dose finding) will recruit patients regardless of their biomarker status. Dose finding in the older age cohort will recruit 3–12 patients (if the younger age cohort is not allowed to open due to too much DLTs in the first 3 patients of the older age cohort, the total number of patients would be 3). If dose finding is initiated in the younger age cohort, it will also require 3–12 patients. This will result in 3–24 patients in total. Patients entering the trial on RP2D in the phase I can seamlessly enter phase II. Patients will be allocated to the four separate cohorts A – D. The sample size in cohorts A, B and C will be restricted by availability of the patients with the biomarkers during the 3-year study recruitment period. The upper limit of patients planned to be recruited to each of the cohorts A, B and C is 20–25. Since the actual patient number is not known exactly, expected type 1 error rate and power are reported averaged over the range of patient numbers to be expected in each cohort. Given the choice of the criteria c_Futility_ and c_Activity_ in the Bayesian adaptive design and assuming that patient numbers between 20 and 25 are equally likely, the expected type 1 error rate is 6.6% and the expected power is 76%. No second interim analysis is planned for cohorts A, B and C. In cohort D recruitment is expected to be faster and the sample size can be determined on the basis of power and 1-sided alpha-level, using the same criteria for futility and efficacy as in cohorts A, B and C. The smallest sample size such that alpha is ≤5% and power ≥ 80% is *n* = 29, for type 1 error is 5.0% and power is 80.3%. The upper limit of patients planned to be recruited to cohort D is therefore 29. A second interim analysis is planned after 20 patients for this cohort. Analytical calculations of the operating characteristics were derived [38] and power calculations were made with the R package BDP2 Version 0.1.3 (from CRAN (https://CRAN.R-project.org/package=BDP2)) which is the core of the web tool BDP2 workflow (http://biostatistics.dkfz.de/BDP2/).

### Statistical methods

#### Primary outcome phase I

A description of safety will be provided on the whole safety population and by dose level. Description of the frequency and percentage of each AE related to the treatment will be provided. RP2D will be defined as the dose level for which at most one out of 6 patients experienced DLT during the priming week and the first cycle, unless otherwise recommended by DMC in collaboration with the coordinating investigator. The steering committee will consider drug related AEs occurring after the first cycle of combination therapy in assessment of overall tolerability.

#### Primary outcome phase II

The activity analysis will be done on the activity population (all patients included in phase II, treated at the RP2D and having received at least one cycle of treatment) and primary analysis will be carried out on each biomarker cohort separately. The final evaluation of activity is based on the same rules for posterior probability as defined for interim analysis.

The estimate of the best response rates (as defined by RECIST or RANO and after central imaging review) will be given with 90% confidence interval in cohorts A, B and C and the estimate of the best response rates will be given with 95% confidence interval in cohort D.

#### Secondary outcomes

(Confirmed immune-related) response will be evaluated separately for CR and PR, and SD and DCR will be evaluated in addition, also using iRECIST and iRANO. DOR will be evaluated for all patients who experienced (confirmed immune-related) response. Starting time point will be the time when best response was determined. The event-time endpoints PFS, OS and TTR will be estimated using the Kaplan-Meier method, considering all the patients who started the treatment, whatever their compliance to treatment, including if the treatment was stopped prematurely for a reason other than disease progression. 95%-confidence intervals will be provided for the Kaplan-Meier estimates.

## Discussion

Pediatric patients with relapsed or refractory disease represent a population with a desperate need for new innovative treatment approaches. The INFORM2 NivEnt trial intends to exploit the immune enhancing effects of entinostat on nivolumab as an innovative new approach. As opposed to the commonly used “all comer” strategy in early phase trials, this trial applies a biomarker-driven approach in order to maximize the chance of detecting early signs of activity. The design allows at the one hand increasing the likelihood of identifying an activity signal by stratifying based on rational biomarkers, while on the other hand it prevents children being exposed to unnecessary risks by early stopping for futility. Early stopping for futility is also applied by other pediatric trials [47]. Large adult initiatives like the National Lung Matrix Trial started to use the Bayesian adaptive design for futility analysis [48]. The Bayesian adaptive design allows more flexibility concerning the number of interims and the final number of patients in each cohort than a frequentist approach [36, 37]. It also opens the possibility to adaptively pool evidence across all cohorts [49] and is therefore applied in the INFORM2 NivEnt trial. In addition, this strategy allows for testing of a new (combination) treatment with relatively brief trial execution timelines and thereby accelerates drug development.

Although the molecular diagnostics typically applied before enrollment [39] are not routinely available in all countries, this is currently changing rapidly in Europe where platforms like the INFORM Registry are becoming available in additional countries every year. For the primary endpoint it could be sufficient to determine only the three relevant biomarkers, but to allow exploration of further potential mechanisms behind responders and non-responders (e.g. potential new biomarkers like immune signatures and infiltration), it is important to have fully molecularly profiled tumors for all patients.

Basket protocols can be challenging due to their complexity, especially when they include adaptive designs. Protocol readability, number and oversight of amendments, sponsor trial oversight, trial governance and a high level of necessary statistical support are well known issues [50]. With four more or less similar arms regarding treatment and statistics in INFORM2 NivEnt, the level of complexity seems justified when considering the acceleration the design offers to drug development in pediatric oncology (with the Bayesian adaptive design also preventing children being exposed to unnecessary risks). By providing the web tool for analytical calculations online (http://biostatistics.dkfz.de/BDP2/), full transparency regarding the Bayesian adaptive design is guaranteed. Subsequent INFORM2 trials following the same robust design are currently in preparation, and will be submitted to the regulatory authorities under a master protocol concept as described before [50]. This will reduce the complexity for investigators, sponsor, statisticians, ethics committees and competent authorities for future INFORM2 trials. In conclusion, the INFORM2 NivEnt biomarker driven basket adaptive design provides an innovative approach accelerating drug development and reducing exposure to investigational treatments in this vulnerable patient population at the same time.

### Trial status

- Protocol version: 9 January 2019, Final2
- Date recruitment start: 26 July 2019
- Estimated recruitment completion: Q3 2021
- Trial registration: ClinicalTrials.gov, NCT03838042. Registered on 12 February 2019.

## Supplementary information

**Additional file 1.** Eligibility criteria

**Additional file 2.** Dose de-escalation table.

**Additional file 3.** Combination treatment modifications.

**Additional file 4.** Visit plan.

## Data Availability

Data sharing is not applicable to this article as no datasets were generated or analyzed yet. However, future pseudonymized trial data will be shared in the context of publications and after publication with other physicians and scientists (national and international academia) to promote and accelerate research on causes and treatment development of oncological diseases.

## Abbreviations

AE: Adverse Event
ADME: Absorption, distribution, metabolism and excretion
ALT: Alanine aminotransferase
AST: Aspartate aminotransferase
CSF: Cerebrospinal fluid
CTCAE: Common Terminology Criteria for Adverse Events
eCRF: Electronic Case Report Form
DLT: Dose Limiting Toxicity
DMC: Data Monitoring Committee
DOR: Duration of response
GPOH: Society of Pediatric Oncology and Hematology
HDAC: Histone deacetylase
HDACi: HDAC-inhibitor
INFORM: INdividualized Therapy FOr Relapsed Malignancies in Childhood
iRANO: Immunotherapy RANO
iRECIST: Immune-related RECIST
ITCC: Innovative Therapies for Children with Cancer
MB: Megabase
PD-L1: Programmed Death-Ligand 1
PDX: Patient derived xenograft
RANO: Response assessment in neuro-oncology
RECIST: Response evaluation criteria in solid tumors
RPKM: Reads Per Million Rotal Reads Per Kilobase of Exon Model
RP2D: Recommended Phase 2 Dose
SNV: Single-nucleotide variant
VHP+: Voluntary Harmonisation Procedure plus
