# Supplementary material for: INFORM2 NivEnt: The first trial of the INFORM2 biomarker driven phase I/II trial series: the combination of nivolumab and entinostat in children and adolescents with refractory high-risk malignancies
Source: BMC Cancer. 2020 Jun 5;20:523. doi: 10.1186/s12885-020-07008-8 (PMC7275428; doi:10.1186/s12885-020-07008-8)
Supplement: Supplementary file 1 — Additional file 1. Eligibility criteria [file 12885_2020_7008_MOESM1_ESM.docx]

**Eligibility criteria**

***Inclusion criteria***

- Children and adolescents with refractory/relapsed/progressive high-risk
  - CNS tumors: medulloblastoma, ependymoma, ATRT, ETMR, pediatric high grade glioma (including DIPG) or other pediatric embryonal CNS tumors

OR

- solid tumors: neuroblastoma, nephroblastoma, rhabdoid tumor, embryonal or alveolar rhabdomyosarcoma or other embryonal small round blue cell tumors including pediatric type (bone) sarcoma

OR

- Children and adolescents with newly diagnosed high grade glioma (HGG) in the context of a constitutional mismatch repair deficiency syndrome after maximum safe surgical resection with no established standard of care treatment option with curative intention available
- No standard of care treatment available
- Age at registration ≥ 6 to ≤ 21 years
- Molecular analysis for biomarker identification (SNV load, PDL1 mRNA expression, MYC/N amplification) in laboratories complying with DIN EN ISO/IEC 17025 or similar via INFORM molecular diagnostic platform or equivalently valid molecular pipeline.
- Biomarker determined using whole exome sequencing (SNV load), RNA-sequencing (PDL1 mRNA expression) and whole genome sequencing (MYC/N amplification).
- In case molecular analysis was not performed via INFORM Registry molecular pipeline: transfer of molecular data (whole exome and RNA sequencing).
- Time between biopsy/puncture/resection of the current refractory/relapsed/progressive tumor and registration ≤ 12 weeks.
- Disease that is measurable as defined by RANO criteria or RECIST v1.1 (as appropriate).
- Life expectancy > 3 months, sufficient general condition score (Lansky ≥ 70 or Karnofsky ≥ 70).
- Transient states like infections can be accepted, and also stable disabilities resulting from disease/surgery (hemiparesis, amputations etc.) can be accepted and will not be considered for Lansky/Karnofsky assessments.
- Laboratory requirements:
  - Hematology: absolute granulocytes ≥ 1.0 × 10^9^/l (unsupported)

platelets ≥ 100 × 10^9^/l

hemoglobin ≥ 8 g/dl or ≥ 4.96 nmol/L

- Biochemistry: Total bilirubin ≤ 1.5 x upper limit of normal (ULN)

AST(SGOT) ≤ 3.0 x ULN

ALT(SGPT) ≤ 3.0 x ULN

serum creatinine ≤ 1.5 x ULN for age

- ECG: normal QTc interval according to Bazett formula < 440ms.
- Patient is able to swallow oral study medication.
- Ability of patient and/or legal representative(s) to understand the character and individual consequences of clinical trial.
- Females of childbearing potential must have a negative serum or urine pregnancy test within 7 days prior to initiation of treatment. Sexually active women of childbearing potential must agree to use acceptable and appropriate contraception during the study and for at least 6 months after the last study treatment administration. Sexually active male patients must agree to use a condom during the study and for at least 7 months after the last study treatment administration.
- Absence of any psychological, familial, sociological or geographical condition potentially hampering compliance with the study protocol and follow-up schedule; those conditions should be discussed with the patient before registration in the trial.
- Before patient screening and registration, written informed consent, also concerning data and blood transfer, must be given according to ICH/GCP, and national/local regulations.
- No prior therapy with the combination of immune checkpoint inhibitors and HDACi.
- BSA ≥ 0.9m^2^
- Phase I: molecular analysis performed and biomarker status known (mutational load, PD-L1 mRNA expression AND MYC(N) amplification status).
- Phase II: molecular analysis performed, biomarker status known (mutational load, PD-L1 mRNA expression AND MYC(N) amplification status) and stratification according to the following criteria:
  - Group A: high mutational load (defined as > 100 somatic SNVs/exome) based on whole exome sequencing

OR

- Group B: high PD-L1 mRNA expression (defined as reads per million total reads per kilobase of exon model (RPKM) > 3) based on RNA sequencing

OR

- Group C: Focal MYC(N) amplification based on whole genome sequencing

OR

- Group D: Patients with biomarker low tumors according to the definitions of group A-C.

***Exclusion criteria***

- Patients with CNS tumors or metastases who are neurologically unstable despite adequate treatment (e.g. convulsions).
- Patients with low-grade gliomas or tumors of unknown malignant potential are not eligible.
- Evidence of > Grade 1 recent CNS hemorrhage on the baseline MRI scan.
- Participants with bulky CNS tumor on imaging are ineligible; bulky tumor is defined as:
  - Tumor with any evidence of uncal herniation or severe midline shift.
  - Tumor with diameter of > 6 cm in one dimension on contrast-enhanced MRI.
  - Tumor that in the opinion of the investigator, shows significant mass effect.
- Previous allogeneic bone marrow, stem cell or organ transplantation.
- Diagnosis of immunodeficiency.
- Diagnosis of prior or active autoimmune disease.
- Evidence of interstitial lung disease.
- Any contraindication to oral agents or significant nausea and vomiting, malabsorption, or significant small bowel resection that, in the opinion of the investigator, would preclude adequate absorption.
- Known history of human immunodeficiency virus (HIV) (HIV 1/2 antibodies). Known active hepatitis B (e.g., hepatitis B surface antigen-reactive) or hepatitis C (e.g., hepatitis C virus ribonucleic acid [qualitative]). Patients with past hepatitis B virus (HBV) infection or resolved HBV infection (defined as the presence of hepatitis B core antibody [HBc Ab] and absence of HBsAg) are eligible. HBV DNA test must be performed in these patients prior to study treatment. Patients positive for hepatitis C virus (HCV) antibody are eligible only if polymerase chain reaction is negative for HCV RNA.
- Clinically significant, uncontrolled heart disease.
- Major surgery within 21 days of the first dose. Gastrostomy, ventriculo-peritoneal shunt, endoscopic ventriculostomy, tumor biopsy and insertion of central venous access devices are not considered major surgery, but for these procedures, a 48 hour interval must be maintained before the first dose of the investigational drug is administered.
- Any anticancer therapy (e.g., chemotherapy, HDACi (including valproic acid), DNA methyltransferase inhibitors, other immunotherapy, targeted therapy, biological response modifiers, endocrine anticancer therapy or radiotherapy) within 4 weeks or at least 5 half-lives (whichever is longer) of study drug administration.
- Radiologically confirmed radiotherapy induced pseudoprogression in CNS tumors.
- Traditional herbal medicines; these therapies are not fully studied and their use may result in unanticipated drug-drug interactions that may cause or confound the assessment of toxicity. As part of the enrollment/informed consent procedures, the patient will be counseled on the risk of interactions with other agents, and what to do if new medications need to be prescribed or if the patient is considering a new over-the-counter medicine or herbal product.
- History of hypersensitivity to the investigational medicinal product or to any drug with similar chemical structure or to any excipient present in the pharmaceutical form (including benzamide) of the investigational medicinal product.
- Participation in other ongoing clinical trials.
- Pregnant or lactating females.
- Presence of underlying medical condition (e.g. gastrointestinal disorders or electrolyte disturbances) that in the opinion of the Investigator or Sponsor could adversely affect the ability of the subject to comply with or tolerate study procedures and/or study therapy, or confound the ability to interpret the tolerability of combined administration of entinostat and nivolumab in treated subjects.
- Patients receiving systemic steroid therapy or any other form of immunosuppressive therapy within 7 days prior to the first dose of study treatment. The use of physiologic doses of corticosteroids (up to 5 mg/m^2^/day prednisone equivalent) may be approved after consultation with the Sponsor.
- No patient will be allowed to enroll in this trial more than once.
