# Supplementary material for: INFORM2 NivEnt: The first trial of the INFORM2 biomarker driven phase I/II trial series: the combination of nivolumab and entinostat in children and adolescents with refractory high-risk malignancies
Source: BMC Cancer. 2020 Jun 5;20:523. doi: 10.1186/s12885-020-07008-8 (PMC7275428; doi:10.1186/s12885-020-07008-8)
Supplement: Supplementary file 2 — Additional file 2. Dose de-escalation table. [file 12885_2020_7008_MOESM2_ESM.docx]

Dose de-escalation.

*Dose level -1 is only applied in the phase II of the trial. If dose-level -1 is also not tolerated, treatment should be discontinued.
