# Supplementary material for: INFORM2 NivEnt: The first trial of the INFORM2 biomarker driven phase I/II trial series: the combination of nivolumab and entinostat in children and adolescents with refractory high-risk malignancies
Source: BMC Cancer. 2020 Jun 5;20:523. doi: 10.1186/s12885-020-07008-8 (PMC7275428; doi:10.1186/s12885-020-07008-8)
Supplement: Supplementary file 3 — Additional file 3. Combination treatment modifications. [file 12885_2020_7008_MOESM3_ESM.docx]

**Combination treatment modifications**

**Combination treatment modifications.**

Modifications always concern the combination treatment and de-escalation shown in Additional file 1.

^a^Recommendation for the use of hormone replacement therapy is provided in the protocol.

^b^Grade 4 drug-related endocrinopathy adverse events, such as, hyper- or hypothyroidism, or glucose intolerance, which resolve or are adequately controlled with physiologic hormone replacement (corticosteroids, thyroid hormones) or glucose-controlling agents, respectively, may not require discontinuation after discussion with and approval from the trail office.

Treatment modification procedures are listed in the above table. The lower grades not listed in this table (mostly Grade 1 and sometimes also Grade 2) do not require a dose modification and can be treated symptomatically, as described in the protocol. Independent of the treatment modifications listed in this table, investigators are responsible for monitoring the degree of burden and associated risks at all time. Treatment should be withheld for any AE, laboratory abnormality, or intercurrent illness which, in the judgment of the investigator, warrants delaying the dose of study medication. Participants who require delay of treatment should be re-evaluated weekly or more frequently if clinically indicated and resume treatment when re-treatment criteria are met.

Grade ≤ 3 neurologic AE consistent with immune-treatment effect (i.e., due to peri-tumoral edema/reaction in brain tumors) and resolved to Grade 0-1 within 14 days (with appropriate treatment) do not need a permanent treatment discontinuation
