# Supplementary material for: INFORM2 NivEnt: The first trial of the INFORM2 biomarker driven phase I/II trial series: the combination of nivolumab and entinostat in children and adolescents with refractory high-risk malignancies
Source: BMC Cancer. 2020 Jun 5;20:523. doi: 10.1186/s12885-020-07008-8 (PMC7275428; doi:10.1186/s12885-020-07008-8)
Supplement: Supplementary file 4 — Additional file 4. Visit plan. [file 12885_2020_7008_MOESM4_ESM.docx]

**Visit plan.**

^a^EOT (end of treatment) is planned as having received 12 cycles/60 weeks of treatment. In individual patients that are experiencing clinical benefit, combination treatment continuation beyond cycle 12 is allowed for a maximum of 12 months or until end of study as described in the protocol. EOT is defined as Day 28 (± 7 days) of the final cycle of treatment (or time of premature discontinuation of treatment).

^b^Unless stated differently.

^c^In case molecular analysis was not performed via the INFORM Registry molecular pipeline, molecular data (whole exome and RNA sequencing) will be transferred in a pseudonymized manner over a secured connection and stored according to relevant privacy and data security regulations.

^d^If a biopsy or resection is performed at progression, a new tumor sample (paraffin tissue section, fresh frozen and viable) should be submitted to INFORM Registry or equivalent molecular pipeline for exploratory analysis of resistance mechanisms.

^e^Maximally 14 days prior to initiation of treatment

^f^Odd cycles only, ± 7 days allowed. In case of treatment delays/pauses, these investigations should not be delayed for more than 4 weeks.

^g^Every 3rd cycle ± 7 days allowed (Cycle 13, 16, 19 and 22). In case of treatment delays/pauses, these investigations should not be delayed for more than 4 weeks.

^h^Assessment should only be performed if no disease assessment was performed in last 2 cycles, including central reference radiology.

^i^No imaging; date of progression (as assessed by treating investigator) and survival status only (in LTFU may be conducted by phone).

^j^Pre-dose and 1 and 6h post dose (please refer to the protocol for maximum allowable volumes in small children). If CSF is being obtained for clinical reasons, please submit CSF for PK analysis.

^k^24h post first dose.

^l^Pre-dose only. If CSF is being obtained for clinical reasons, please submit CSF for PK analysis.

^m^Maximally 48 hours before next drug dose, if applicable.

^n^Medication may be administered ± 48 hours the scheduled dose. Nivolumab administration defines Day 1 of a cycle, entinostat should be scheduled accordingly.

-For blood sample priorities in small children, please refer to the protocol

-Abbreviations: D; day of respective cycle, FUP; follow-up, LTFU; long-term follow-up, WOCBP; women of childbearing potential, ctDNA; circulating tumor DNA
